# Voxel-Based Morphometry Correlates of an Agitated-Aggressive Syndrome in the At-Risk Mental State for Psychosis and First Episode Psychosis

**DOI:** 10.1038/s41598-018-33770-8

**Published:** 2018-11-08

**Authors:** Christian G. Huber, Sonja Widmayer, Renata Smieskova, Laura Egloff, Anita Riecher-Rössler, Rolf-Dieter Stieglitz, Stefan Borgwardt

**Affiliations:** 1 Department of Psychiatry, University Hospital Basel, University of Basel, Basel, Switzerland; 2 Psychological Faculty, University of Basel, Basel, Switzerland

## Abstract

There are mixed reports on structural neuroimaging correlates of aggression in schizophrenia with weak evidence due to cohort overlaps and lack of replications. To our knowledge, no study examined volumetric neuroimaging correlates of aggression in early stages of psychosis. An agitated-aggressive syndrome is present in at-risk mental state (ARMS) and in first-episode psychosis (FEP) – it is unclear whether this syndrome is associated with structural brain abnormalities in early stages of psychosis. Using three-dimensional magnetic resonance imaging and a whole brain voxel-based morphometry approach, we examined 56 ARMS patients, 55 FEP patients and 25 healthy controls. We operationalized aggression using the Excited Component of the Brief Psychiatric Rating Scale (BPRS-EC) and dichotomized our patient group by median split into “BPRS-EC high” (n = 49) and “BPRS-EC low” groups (n = 62). The “BPRS-EC high” group had significantly smaller left lingual gyrus volume than HC. This finding was not present in the “BPRS-EC low” group. In addition, grey matter volume in the left lingual gyrus showed a negative linear correlation with BPRS-EC over all subjects (*ρ* = −0.318; *p* = 0.0001) and in the patient group (*ρ* = −0.202; *p* = 0.033). These findings provide first hints on structural brain abnormalities associated with an agitated-aggressive syndrome in ARMS and FEP patients.

## Introduction

In first-episode psychosis, there is a prevalence of violence in 34.5% and a prevalence of serious violence in 16.6% of cases with the duration of untreated psychosis being an important influencing factor^1^. Violence in this patient group poses severe clinical problems and a challenge for patients, relatives and professionals^2^. The Brief Psychiatric Rating Scale-Excited Component (BPRS-EC) and neuropsychological dysfunction have been shown to predict aggression in first-episode psychosis^3^. We showed earlier that an agitated-aggressive syndrome was already present in at-risk mental state (ARMS) for psychosis and first-episode psychosis (FEP)^4^. Also, there is evidence for an agitated-aggressive syndrome in early-onset psychosis^5^. However, it is not clear whether this agitated-aggressive syndrome is associated with structural brain abnormalities in early stages of psychosis.

Independently of correlates with aggression, there are consistent grey matter (GM) reductions both in ARMS and FEP patients when compared to healthy controls (HC)^6^. Fusar-Poli *et al*. reported GM reductions in the temporal, limbic prefrontal cortex in the ARMS group, and in the temporal insular cortex and cerebellum in the FEP group^6^. Psychosis onset was characterized by GM decreases in temporal, anterior cingulate, cerebellar, and insular regions. Furthermore, GM alterations in the temporal regions were directly related to the severity of psychotic symptoms^6^.

There are mixed results on structural neuroimaging correlates of aggression in schizophrenia^7–9^. Most studies operationalized aggression as “history of violence”, while others used continuous measures of aggression. In general, findings pointed to decreased brain volumes in aggressive versus non-aggressive schizophrenia patients (e.g. decreased volumes in cerebellum, prefrontal cortex, premotor cortex, inferior frontal cortex and hippocampus)^10–15^, while others reported a volume increase in specific structures (e.g., increased amygdala volumes in violent schizophrenia patients^11^), or no significant differences in brain volumes^16^. A recent overview on structural magnetic resonance imaging correlates of aggression in psychosis can be found in Widmayer *et al*.^17^.

More specifically, schizophrenia patients with a history of violence had a significantly reduced whole-brain volume compared to schizophrenia patients without a history of violence^10^. The violent schizophrenia group showed a significantly larger putamen volume than the non-violent group; also, in that group comparison, amygdala volume was found to be reduced – these findings, though, were not sustained when *Positive and Negative Syndrome Scale* (PANSS) general psychopathology score was used as covariate. Subjects with schizophrenia and a history of violence had smaller whole brain, temporal lobe and hippocampus volumes than schizophrenia patients without a history of violence^11^. However, schizophrenia patients with a history of violence had larger amygdala volumes than the schizophrenia patients without a history of violence. When comparing the anterior cingulate volumes of violent with non-violent patients, they did not differ significantly^16^. Aggressive versus non-aggressive schizophrenia patients exhibited reduced cortical thickness in ventromedial prefrontal and lateral sensorimotor cortex especially in the right hemisphere^15^. All papers mentioned in the above paragraph based their examinations on the same patient sample.

Another study reported reduced grey matter volume in whole brain, hippocampus and parahippocampal gyrus in violent as compared to non-violent schizophrenia patients^12^. Also, violent schizophrenia patients had smaller grey matter volumes in the cerebellum than non-violent schizophrenia patients^13^.

In two papers, dimensional measures of aggression were used to examine structural correlates of violence in one sample of schizophrenia patients^18,19^. The first found larger grey matter volumes in the left orbitofrontal cortex to be associated with a higher degree of aggression as rated using the PANSS and the *Overt Aggression Scale* (OAS)^18^, while the other reported larger caudate volumes in patients with treatment-resistant schizophrenia^19^.

Summing up, there are relatively few and partly contradictory reports on neuroimaging correlates of aggression in schizophrenia, while inconsistent operationalization of aggression, extensive cohort overlaps and lack of replication studies complicate the interpretation and synthesis of the findings. Furthermore, to our knowledge, no study has examined neuroimaging correlates of aggression in the early stages of psychosis.

Therefore, we aimed at characterizing regions where grey matter volume (GMV) is associated with an agitated-aggressive syndrome in early psychosis using voxel-based morphometry (VBM). Because an agitated-aggressive syndrome as measured with the BPRS-EC had previously been reported in ARMS and FEP patients, and BPRS-EC has been shown to predict clinical aggression, we chose to operationalize aggression using this instrument. Furthermore, because none of the previous findings on GMV correlates of aggression in psychosis has been successfully replicated so far, we chose an exploratory whole brain magnetic-resonance imaging (MRI) approach. Based on previous literature, we hypothesized that a subgroup of ARMS and FEP patients would present with an agitated-aggressive syndrome, and that this subgroup would show reduced GMV in brain regions associated with aggression.

## Results

### Demographics and Clinical Group Differences

As presented in Table 1, there were significant gender differences with an overrepresentation of male participants in the ARMS and FEP patient groups. The groups did not differ significantly in age, but the patient groups showed a significantly lower level of education than the healthy controls.

**Table 1:** Demographics and Clinical Group Differences.

	BPRS-EC high (H) (n = 49)			BPRS-EC low (L) (n = 62)			HC (C) (n = 25)	Statistics (H vs. L vs. C)	Post hoc
	ARMS	FEP	Total	ARMS	FEP	Total			
Gender M/F	13/7	24/5	37/12	28/8	16/10	44/18	11/14	χ^2^(2) = 8.8 *p* = 0.018	
Mean age (years) mean (SD)	23.8 (4.1)	26.7 (6.9)	25.5 (6.1)	24.7 (6.0)	28.0 (7.9)	26.1 (7.0)	27.7 (4.5)	F(2,1) = 0.959 *p* = 0.386	
Years of education mean (SD)	13.8 (2.4)	12.6 (3.2)	13.1 (2.9)	13.4 (3.1)	13.7 (2.6)	13.5 (2.9)	16.0 (3.1)	F(2,1) = 6.9 *p* = 0.001	H < C, L < C
BPRS total mean (SD)	43.6 (9.4)	56.9 (11.3)	52.3 (11.5)	35.7 (6.4)	42.3 (11.2)	38.8 (9.5)	24.5 (1.1)	F(2,1) = 70.4 *p* < 0.001	H > C, H > L, L > C
BPRS-EC mean (SD)	7.1 (1.6)	8.3 (2.4)	7.8 (2.2)	4.4 (0.5)	4.2 (0.4)	4.3 (0.5)	4.0 (0.0)	F(2,1) = 113.2 *p* < 0.001	H > C, H > L
SANS total Mean (SD)	21.7 (17.1)	25.6 (14.4)	24.1 (15.5)	15.2 (10.7)	18.8 (16.3)	16.8 (13.4)	0.0 (0.0)	F(2,1) = 26.9 *p* < 0.001	H > C, H > L, L > C
GAF total Mean (SD)	66.9 (10.7)	54.7 (15.1)	59.8 (14.6)	67.8 (12.2)	60.3 (13.8)	64.6 (13.3)	88.4 (4.4)	F(2,1) = 43.9 *p* < 0.001	H < C, L < C
Antipsychotics n (%)	0	12 (24.5%)	12 (24.5%)	0	12 (19.4%)	12 (19.4%)	0	χ^2^(1) = 0.0 *p* = 0.939	
Antidepressants n (%)	12 (24.5%)	6 (12.3%)	18 (36.7%)	10 (16.1)	4 (6.4)	14 (22.6%)	0	χ^2^(1) = 5.5 *p* = 0.188	
Alcohol n No/Mod/Uncon	6/10/3	11/15/3	17/25/6	6/25/5	8/15/3	14/40/8	1/22/2	χ^2^(4) = 10.6 *p* = 0.032	
Cannabis currently n (%)	8 (16.3%)	9 (18.4%)	17 (34.7%)	9 (14.5%)	5 (8.1%)	14 (22.6%)	4 (16%)	χ^2^(2) = 3.8 *p* = 0.152	
Smoking cig/day mean (SD)	11.9 (9.1)	10.6 (8.7)	11.1 (8.8)	6.9 (9.3)	12.3 (13.3)	9.2 (11.3)	3.1 (6.4)	F(2,1) = 5.7 *p* = 0.004	H > C, L > C

Bonferroni correction (at *p* < 0.05) was calculated for *post-hoc* analyses in SPSS 25.0. Abbreviations: BPRS-EC high: individuals prone to psychosis with high BPRS Excited Component score defined as BPRS-EC > 5, listed as H in the Statistics and Post hoc column; BPRS-EC low: individuals prone to psychosis with low BPRS Excited Component score defined as BPRS-EC ≤ 5, listed as L in the Statistics and Post hoc column; HC: healthy controls, listed as C in the Statistics and Post hoc column; BPRS total: Brief Psychiatric Rating Scale total score; BPRS-EC: Brief Psychiatric Rating Scale – Excited Component; SANS total: Scale for the Assessment of Negative Symptoms total score; GAF total: Global Assessment of Functioning total score; Alcohol n = number of subjects consuming alcohol; No: no alcohol; Mod: moderate intake of alcohol; Uncon: uncontrolled drinking; Smoking cig/day: amount of cigarettes smoked per day.

In our clinical measures, patients showed a significantly higher BPRS total score than healthy controls. Also, the “BPRS-EC high” group exhibited a significantly higher BPRS total score than the “BPRS-EC low” group. BPRS-EC was significantly higher in patients than in healthy controls and higher in the “BPRS-EC high” group than in the “BPRS-EC low” group. *Scale for the Assessment of Negative Symptoms* (SANS) total score was significantly higher in patients than in healthy controls, with the “BPRS-EC high” group showing significantly higher scores than the “BPRS-EC low” group. Both patient groups had a significantly lower *Global Assessment of Functioning* (GAF) total score than healthy controls.

Regarding medication, our patient groups did not differ significantly in the intake of antipsychotic medication, but a significantly higher proportion of the “BPRS-EC high” group were under antidepressant pharmacotherapy than of the “BPRS-EC low” group. At the time of scanning, 12 individuals were medicated with low doses of atypical antipsychotic medication (7 in the “BPRS-EC low” and 5 in the “BPRS-EC high” group), while 28 individuals received antidepressants (11 in the “BPRS-EC low” and 17 in the “BPRS-EC high” group).

With respect to substance use, our groups did not differ significantly in consumption of cannabis, but patients smoked significantly more cigarettes and had significantly increased moderate and uncontrolled alcohol intake than healthy controls.

Table 2 shows the correlations of the BPRS-EC and its items with BPRS total score, BPRS total score without items included in the BPRS-EC, and SANS total. There were weak to moderate positive correlations between the BPRS-EC items *hostility*, *tension*, *uncooperativeness* and *excitement* and BPRS total score without BPRS-EC items, and weak correlations of most BPRS-EC items and BPRS-EC score with SANS total score.

**Table 2:** Correlations of BPRS-EC items and total score with BPRS total score, BPRS total score without BPRS-EC items, and SANS total score.

		Hostiliy	Tension	Uncooperativeness	Excitement	BPRS-EC
BPRS total	r	0.475**	0.537**	0.395**	0.412**	0.705**
	*p*	<0.001	<0.001	<0.001	<0.001	<0.001
	N	124	124	124	124	124
BPRS total without BPRS-EC items	r	0.367**	0.501**	0.229**	0.392**	0.591**
	*p*	<0.001	<0.001	0.007	<0.001	<0.001
	N	136	136	136	136	136
SANS total	r	0.229*	0.238**	0.212**	0.124	0.325**
	*p*	0.011	0.008	0.008	0.176	<0.001
	N	121	121	121	121	121

*Note*. **Correlation is significant at *p* < 0.01 (2-tailed). *Correlation is significant at *p* < 0.05 (2-tailed). r: Pearson’s correlation; *p:* two-tailed significance level. BPRS total: Brief Psychiatric Rating Scale total score; BPRS-EC: Brief Psychiatric Rating Scale – Excited Component; SANS total: Scale for the Assessment of Negative Symptoms total score.

In Table 3, we show the correlations between the BPRS-EC items and BPRS-EC score with the other BPRS items. The individual BPRS items not included in the BPRS-EC showed several very weak to weak positive correlations with the BPRS-EC items and total score. Only anxiety (vs. tension and BPRS-EC), grandiosity (vs. BPRS-EC), suspiciousness (vs. tension and BPRS-EC), and unusual thought content (vs. tension and BPRS-EC) showed positive correlations of moderate strength.

**Table 3:** Correlations of BPRS-EC items and total score with the remaining BPRS items.

		Hostiliy	Tension	Uncooperativeness	Excitement	BPRS-EC
Somatic concern	r	0.133	0.162	0.130	0.179*	0.215*
	*p*	0.124	0.059	0.132	0.037	0.012
	N	136	136	136	136	136
Anxiety	r	0.201*	0.523**	0.109	0.346**	0.497**
	*p*	0.019	<0.001	0.207	<0.001	<0.001
	N	136	136	136	136	136
Depression	r	0.166	0.283**	0.251**	0.098	0.310**
	*p*	0.054	0.001	0.003	0.256	<0.001
	N	136	136	136	136	136
Suicidality	r	0.274**	0.182*	0.247**	0.207*	0.354**
	*p*	0.001	0.034	0.004	0.016	<0.001
	N	136	136	136	136	136
Guilt	r	0.203*	0.248**	0.163	0.173*	0.301**
	p	0.018	0.004	0.058	0.044	<0.001
	N	136	136	136	136	136
Elevated mood	r	0.167	0.102	0.250**	0.232**	0.246**
	*p*	0.052	0.239	0.003	0.006	0.004
	N	136	136	136	136	136
Grandiosity	r	0.275**	0.291**	0.319**	0.307**	0.427**
	*p*	0.001	0.001	<0.001	<0.001	<0.001
	N	136	136	136	136	136
Suspiciousness	r	0.396**	0.441**	0.090	0.342**	0.558**
	*p*	<0.001	<0.001	0.299	<0.001	<0.001
	N	136	136	136	136	136
Hallucinations	r	0.230**	0.377**	−0.037	0.244**	0.373**
	*p*	0.007	<0.001	0.668	0.004	<0.001
	N	136	136	136	136	136
Unusual thought content	r	0.322**	0.406**	0.075	0.395**	0.482**
	*p*	<0.001	<0.001	0.387	<0.001	<0.001
	N	136	136	136	136	136
Bizarre behavior	r	0.181*	0.337**	0.067	0.208	0.338**
	*p*	0.035	<0.001	0.440	0.015	<0.001
	N	136	136	136	136	136
Self-neglect	r	0.066	0.261**	0.073	0.047	0.209*
	*p*	0.448	<0.001	0.397	0.583	0.015
	N	136	136	136	136	136
Disorientation	r	0.025	−0.085	−0.034	−0.040	−0.036
	*p*	0.776	0.323	0.695	0.642	0.677
	N	136	136	136	136	136
Conceptual disorganization	r	0.224**	0.125	−0.023	0.114	0.223**
	*p*	0.009	0.146	0.793	0.188	0.009
	N	136	136	136	136	136
Blunted affect	r	0.204*	0.168	0.139	0.221*	0.317**
	*p*	0.017	0.051	0.106	0.010	<0.001
	N	136	136	136	136	136
Emotional withdrawal	r	0.108	0.202*	0.099	0.011	0.218*
	*p*	0.210	0.019	0.254	0.898	0.011
	N	136	136	136	136	136
Motor retardation	r	0.126	−0.062	0.123	0.022	0.047
	*p*	0.142	0.473	0.152	0.795	0.585
	N	136	136	136	136	136
Distractibility	r	0.125	0.225**	0.172*	0.238**	0.249**
	*p*	0.146	0.009	0.045	0.005	0.004
	N	136	136	136	136	136
Motor hyperactivity	r	−0.041	0.237**	−0.037	0.320**	0.167
	*p*	0.637	0.005	0.667	<0.001	0.051
	N	136	136	136	136	136
Mannerism and posturing	r	0.090	0.253**	0.220*	0.022	0.174*
	*p*	0.298	0.003	0.010	0.802	0.043
	N	136	136	136	136	136

*Note* **Correlation is significant at *p* < 0.01 (2-tailed). *Correlation is significant at *p* < 0.05 (2-tailed). r: Pearson’s correlation; *p:* two-tailed significance level. BPRS: Brief Psychiatric Rating Scale; BPRS-EC: Brief Psychiatric Rating Scale – Excited Component.

### Imaging Results

**Figure 1:**
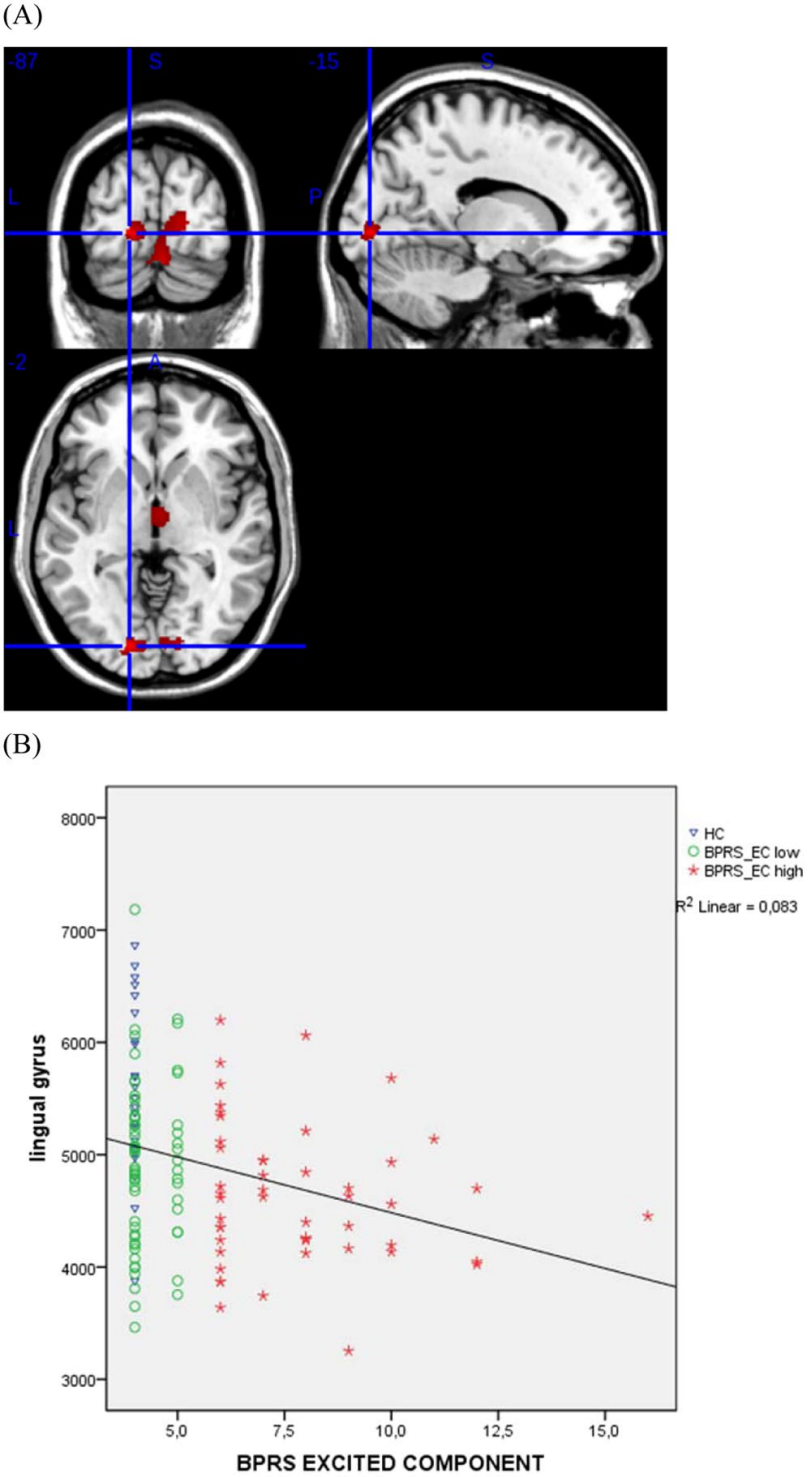
Correlation of BPRS-EC and Lingual Gyrus Volume. Correlation of BPRS-EC and grey matter volume (GMV) in the lingual gyrus. (**A**) The cluster in the lingual gyrus (MNI x, y, z = −15, −87, −2) reflects reduced GMV in the “BPRS-EC high” group compared to the healthy control group [*p* < 0.05 family-wise error (FEW) corrected]. (**B**) Correlation of BPRS-EC and lingual gyrus GMV in this cluster.

As shown in Fig. 1, the “BPRS-EC high” group had significantly less GMV in the left lingual gyrus as compared to HC. Statistical threshold was *p* < 0.05 after family-wise error (FWE) correction. There were no significant between-group differences regarding the contrasts “BPRS-EC high” >HC, “BPRS-EC high” > “BPRS-EC low”, “BPRS-EC high” < “BPRS-EC low”, patients > HC, and patients < HC.

To better understand the relationship between the GMV abnormalities and the BPRS-EC we performed correlation analyses. A negative correlation was detected between GMV in the left lingual gyrus and BPRS-EC score (MNI x, y, z = −15, −87, −2; Fig. 1) in all included individuals (*ρ* = −0.318; *p* = 0.0001) and in the patient group (ARMS & FEP; *ρ* = −0.202; *p* = 0.033).

## Discussion

Aiming at investigating grey matter correlates of aggression, we dichotomized individuals in early stages of psychosis according to BPRS-EC into a “BPRS-EC high” and “BPRS-EC low” group. The BPRS-EC mean value did not differ significantly between the “BPRS-EC low” group and healthy controls.

Our voxel-based morphometry study showed that individuals in early stages of psychosis with an agitated-aggressive syndrome have significant volumetric reductions in the lingual gyrus as opposed to healthy control participants. These volumetric reductions were not evident when comparing BPRS-EC high versus BPRS-EC low groups. This could reflect that a reduced lingual gyrus volume may not exclusively be related to an agitated-aggressive syndrome, but may also in part be a common disease-related correlate of early psychosis – thereby, volumetric alterations could also be present in ARMS and FEP without an agitated-aggressive syndrome to some degree. Furthermore, treatment with antipsychotic medication may already influence potential differences in brain volumes in this early stage of psychosis^20,21^. Longitudinal studies may provide better hints towards an understanding of the specificity of the lingual gyrus volume reduction in aggressive behaviour in schizophrenia.

Lingual gyrus reductions were already described in first-episode psychosis individuals^22,23^ – however, the behavioural correlate of this volumetric reduction was unclear. Furthermore, we observed a negative correlation between lingual gyrus volume and BPRS-EC. It has been reported that aggression in psychoses operationalized by dimensional measures (i.e., the PANSS items “Hostility” and “Poor Impulse Control”^24^ and the OAS) was associated with larger grey matter volumes in the left orbitofrontal cortex and also larger caudate volumes^18,19^. Our findings might be different because we used an alternative operationalization of aggression. Also, our participants were ARMS and FEP patients compared to the treatment-resistant schizophrenia patients examined by Hoptman *et al*.^18,19^. There are no other studies on structural MRI correlates of aggression in early stages of psychoses. Independently of a psychotic disorder, though, a study examined structural correlates of aggression in patients with borderline personality disorder and found that high as opposed to low lethality suicide attempters had diminished grey matter in an extensive fronto-limbic network including the left lingual gyrus^25^. The authors discuss that deficits in this network could impair social functioning^25^. In studies about correlates of aggression in healthy persons, the lingual gyrus does not seem to be altered.

When looking at functional correlates of aggression in the lingual gyrus, though, we find hints that the region may play an important role. For example, activation to aggressive stimuli in spouse abusers revealed that batterers, relative to controls, showed less activation in the left lingual gyrus when responding to aggressive words^26^. In patients with psychoses, there are two studies reporting functional correlates of aggression in the lingual gyrus. In the first study, participants were threatened to receive an electric shock – when anticipating this shock, violent persons with schizophrenia as opposed to healthy controls showed hyperactivation in the right lingual gyrus^27^. In another study, authors showed negative emotional pictures to their participants and observed that violent persons with schizophrenia as opposed to healthy controls showed significant hyperactivations in the left lingual gyrus^28^. Also, violent as opposed to non-violent persons with schizophrenia showed hyperactivations in response to negative emotional pictures in the right lingual gyrus^28^.

The substantial differences in operationalization of aggression and sample composition make studies difficult to compare, and replication studies are needed in order to further evaluate structural correlates of aggression in psychoses.

While there are many results indicating an important role of the lingual gyrus in aggression, it remains unclear exactly how this early volumetric abnormality is associated with aggressive behaviour in early stages of psychoses. Still, our results support the hypothesis that there is, indeed, a structural correlate to an agitated-aggressive syndrome in very early psychoses that is potentially linked to a differential processing of negative emotions.

Some limitations of the current study have to be considered: First, our patient groups differed significantly in the intake of antidepressants and the consumption of nicotine; as we examined an adequate but still small sample, we could not correct for all potential influencing factors in our comparisons. Therefore, we cannot exclude that parts of the reported differences in brain volume may have been affected by substance use. In addition, some potential influencing variables of aggression (e.g., forensic history, antisocial personality disorder) were not available for analysis.

Furthermore, dichotomizing the patient group using a median split may not be an ideal approach, but is a method often used when there is no clear cutoff for clinical relevance, as it is the case with the BPRS-EC. According to the median split, patients with BPRS-EC scores from 4 to 5 were entered in the “BPRS-EC low” subgroup, and patients with BPRS-EC scores from 6 on upwards in the “BPRS-EC high” subgroup. A score of 4 corresponds to complete absence of agitation and aggression (all four items rated as *absent*), and a score of 5 to nearly complete absence with three items rated as *absent*, and one item rated as *very mild*. All control participants in the current study had BPRS-EC scores of 4. Therefore, the median split corresponds to a dichotomization into a group with near complete absence and with the presence of an agitated-aggressive syndrome.

In addition, the “BPRS high” and “BPRS low” subgroups also significantly differed in BPRS and SANS total scores, and it is known that psychopathological symptoms are often interrelated: correlation analyses in our sample showed very weak to moderate correlations between most BPRS-EC items, BPRS items not included in the BPRS-EC, and SANS total score. This raises the question how specific the results are for BPRS-EC. However, the collinearity of BPRS-EC, BPRS total score, and SANS total score also poses a methodological challenge, as controlling for the effect of BPRS total score in our analyses could also diminish or remove true positive findings of significant associations with BPRS-EC. Indeed, exploratory additional VBM analyses in CAT12 (http://www.neuro.uni-jena.de/cat/) including a BPRS sum score without the items present in the BPRS-EC as a covariate did not show significant results any more, but it is unsure how this should be interpreted. In line with the hypothesis presented above, inclusion of the additional BPRS items could lead to a false negative result, and furthermore, the study may be underpowered for this analysis. Concerning the association between BPRS-EC and the SANS, lingual gyrus volume has until now not been shown to correlate with negative symptoms in schizophrenia^29^. This strengthens the hypothesis that the reduced lingual gyrus volume found in the “BPRS high” group is not associated with increased negative symptoms in this group. Therefore, the presented findings constitute first evidence for a structural correlate of an agitated-aggressive syndrome in ARMS and FEP, but have to be replicated in further studies.

Additionally, the analysis strategy of combining ARMS and FEP patients could be questioned. From the authors’ point of view, this decision is warranted because an agitated-aggressive syndrome is already present in ARMS patients^4^ and because there is strong evidence supporting a continuum model of psychotic symptoms^30^. However, the theoretical possibility exists that the findings could predominantly be driven by more acutely ill FEP patients and could not be generalizable to ARMS. Again, exploratory additional VBM analyses in CAT12 (http://www.neuro.uni-jena.de/cat/) including a diagnostic group as a covariate did not show significant results any more, but – as noted above – the interpretation of this finding remains unsure with respect to the limited sample size. Although FEP patients showed considerably higher total BPRS total scores than ARMS patients – as has to be expected from the diagnostic and inclusion criteria – this was not the case for BPRS-EC, where the differences between diagnostic groups within the “BPRS-EC high” (7.1 vs. 8.3) and “BPRS-EC low” (4.4 vs. 4.2) groups were small to negligible. In addition, although the percentage of FEP patients in the “BPRS-EC high” group was higher compared to the “BPRS-EC low group” (59.2% vs. 41.9%), this difference also seems small considering the potential systematic effect of the diagnostic criteria. Again, the current study has to be considered a pilot study presenting first evidence for a structural correlate of an agitated-aggressive syndrome in ARMS and FEP, and replication studies are needed.

Lastly, the results would not hold if an initial peak-level threshold of *p* < 0.001 had been chosen. Together with the ongoing discussion about the possible inflation of false positive results due to cluster-level corrections^31^ and the controversy on whether or not to use VBM^32^ – particularly when examining small samples – this is a further limitation of the current study.

Still, our findings constitute a first hint that the left lingual gyrus GMV may be inversely correlated with an agitated-aggressive syndrome in early stages of psychoses. If this finding could be reliably replicated it may constitute the first step in translational research towards formulation of specific biological hypotheses on the nature of aggressive behaviour in early stages of psychoses and in the prevention of aggressive behaviour in schizophrenia.

## Materials and Methods

### Study Sample

The current analyses are based on data from the early detection of psychosis project (FePsy) at the Department of Psychiatry, University of Basel, Switzerland^4,33,34^. ARMS patients, FEP patients, and HC for the current analyses were included from November 2008 to April 2014. We identified the patient groups using the criteria of Yung *et al*.^35^ – a detailed description of the study design can be found in Riecher-Rössler *et al*.^33^.

The inclusion criteria for the ARMS group (n = 56) were one or more of the following: (a) “attenuated” psychotic symptoms; (b) brief limited intermittent psychotic symptoms; (c) a first-degree relative with a psychotic disorder plus a marked decline in social or occupational functioning; or (d) unspecific risk category^33,34,36^. Risk assessment was performed with the Basel Screening Instrument for Psychosis (BSIP) specifically designed for early stages of psychosis^37^. After the baseline assessment, the ARMS subjects were followed up clinically and received standard psychiatric case management. 13 out of 56 included ARMS individuals made the transition to psychosis (23% transition rate).

FEP patients (n = 55) fulfilled criteria for acute psychotic disorder according to the ICD-10 or DSM-IV. Brief Psychiatric Rating Scale (BPRS) ratings were conducted by trained raters during the clinical interviews, and all information available (chart reviews, and third-party accounts where applicable) was used in the assessment. Inclusion required ≥4 on the *hallucination* item or ≥5 on the *unusual thought content*, *suspiciousness* or *conceptual disorganization* items of the BPRS^35^, with symptoms occurring at least several times a week and persisting for more than one week. These inclusion criteria predominantly identify patients with a first episode of schizophrenia (F20.x), delusional disorder (F22.x), acute and transient psychotic disorder (F23.x), schizoaffective disorder (F25.x), and other (F28.x) and unspecified (F29.x) nonorganic psychotic disorder^33^.

As an agitated-aggressive syndrome is already present in ARMS and FEP patients^4^, we chose not to perform separate analyses for FEP and ARMS patients, but to examine them as one patient group. For the analysis related to the agitated-aggressive syndrome we dichotomised this patient group according to BPRS-EC using a median split (median_BPRS-EC_ = 5). We then labelled patients with a BPRS-EC score >5 as the “BPRS-EC high” (n = 49) subgroup and patients with a BPRS-EC score ≤5 as the “BPRS-EC low” (n = 62) subgroup.

We recruited healthy volunteers (HC, n = 25) from the same geographical area as the clinical groups. The healthy controls had no current psychiatric disorder, no history of psychiatric illness, head trauma, neurological illness, serious medical or surgical illness, substance abuse and no family history of any psychiatric disorder as assessed by an experienced psychiatrist in a detailed clinical assessment. Data are available on request.

We applied the following exclusion criteria to our patient groups: history of previous psychotic disorder; psychotic symptomatology secondary to an ‘organic’ disorder; recent substance abuse (exception: cannabis) according to ICD-10 research criteria; psychotic symptomatology associated with an affective psychosis or a borderline personality disorder; age <18 years; inadequate knowledge of the German language; and IQ <70 as measured with the multiple choice word test (MWT-B)^38^.

The study was approved by the local research ethics committee of the University of Basel, *Ethikkommission Nordwest- und Zentralschweiz EKNZ*, and all participants provided written informed consent. The authors assert that all procedures contributing to this work comply with the ethical standards of the relevant national and institutional committees on human experimentation and with the Helsinki Declaration of 1975, as revised in 2008.

### Clinical Assessment Scales

The Positive and Negative Syndrome Scale for Schizophrenia (PANSS) Excited Component (PANSS-EC) has been identified as a stable factor in patients with schizophrenia spectrum disorders, but item composition may differ depending on the examined study population^2^. The original PANSS-EC proposed by Lindenmayer *et al*.^39^ consisted of the items *uncooperativeness*, *poor impulse control*, *excitement*, and *hostility*, and did not include the item *tension* due to moderate factor loading^39^. However, PANSS-EC subscales comprising the five items *poor impulse control*, *tension*, *hostility*, *uncooperativeness*, and *excitement* have also been repeatedly identified^2^. In analogy, BPRS-EC subscales have been constructed encompassing the items *excitement*, *hostility*, and *uncooperativeness* with^4^ or without the item *tension*^5^. In the current study, to ensure comparability with previous studies in patients with ARMS and recent onset psychosis, we assessed subjects using the BPRS-EC containing the items *excitement*, *hostility*, *uncooperativeness* and *tension*^3^. Also, subjects were assessed with the SANS^40^ and GAF^41^ at the time of scanning. Additionally, we obtained current and previous alcohol, nicotine, cannabis and other illegal drug consumption using a semi-structured interview adapted from the Early Psychosis Prevention and Intervention Centre (EPPIC) Drug and Alcohol Assessment Schedule (http://www.eppic.org.au).

### Structural Magnetic Resonance Image Acquisition

We acquired a three-dimensional T1-weighted magnetization prepared rapid gradient echo (MPRAGE) sequence on a 3-T MRI system (Magnetom Verio, Siemens Healthcare, Germany) with sagittal orientation based on a 256 × 256 × 176 matrix, with 1 mm isotropic spatial resolution, inversion time (TI) of 1000 ms, repetition time (TR) of 2 s and echo time (TE) of 3.4 ms. An experienced neuroradiologist screened the scans for gross radiological abnormalities.

### Image Analysis

We used SPM8 software (http://www.fil.ion.ucl.ac.uk/spm; Wellcome Department of Cognitive Neurology, UK) running under Matlab 7.1 (MathWorks, USA) to identify group-related differences in grey matter volume (GMV). Voxel-based morphometry was performed using the VBM8 toolbox (http://dbm.neuro.uni-jena.de/vbm8/; earlier VBM analyses with the sample are reported in Smieskova, Borgwardt *et al*.^30,42,43^). T1-weighted MPRAGE images were co-registered to the Montreal Neurological Institute (MNI) template using a multiple stage affine transformation with 12 estimated parameters of interest. These normalized images were segmented using the New Segmentation approach with different treatment of the mixing proportions. Afterwards the changes in volume induced by normalization were corrected using the DARTEL toolbox to produce a high-dimensional normalization protocol^31^. We smoothed all preprocessed images using an isotropic 8 mm Gaussian kernel. We then identified five subjects with a mean covariance below two standard deviations and screened their volumes thoroughly: We found no artefacts and an adequate quality of images. We therefore decided to continue the statistical analysis with all included subjects.

We performed an analysis of covariance (ANCOVA) to compare grey matter images between our three groups (“BPRS-EC high”, “BPRS-EC low” and HC) in the whole brain using voxel based morphometry. We modelled age, gender and total intracranial volume (ICV) as covariates of no interest to reduce the potential impact of these variables on the findings. Statistical significance was assessed at cluster level at a threshold of p < 0.005, uncorrected (cluster-forming threshold) and inferences were made at p < 0.05 after family-wise error (FWE) correction. The eigenvariates from between-group contrasts were extracted and used for correlation analyses between grey matter volume by agitated-aggressive syndrome score.

### Statistical Analyses of Demographics and Clinical Group Differences

We performed ANOVAs and χ^2^-tests to describe group characteristics with regard to gender, age, years of education, BPRS total score and BPRS-EC, SANS total score, GAF score, intake of antipsychotics and antidepressants, as well as consumption of alcohol, cannabis and cigarettes. ANOVAs were followed-up using post-hoc Bonferroni analyses to identify subgroup differences. Furthermore, we calculated Pearson’s correlations for BPRS-EC items and BPRS-EC with BPRS total score, SANS total score, and the BPRS items not included in the BPRS-EC. Correlation strength was assessed as very weak (r < 0.200), weak (0.200 ≤ r < 0.400), moderate (0.400 ≤ r < 0.600), strong (0.600 ≤ r < 0.800), and very strong (r ≥ 0.800) as recommended by Evans (1996)^44^. All analyses were performed with the “Statistical Package for Social Sciences” (IBM SPSS Statistics 25.0), and *p* < 0.05 was considered as significant.

## References

[1] Large MM, Nielssen O (2011). Violence in first-episode psychosis: a systematic review and meta-analysis. Schizophrenia Research.

[2] Huber CG, Naber D, Lambert M (2008). Incomplete remission and treatment resistance in first-episode psychosis: definition, prevalence and predictors. Expert Opinion on Pharmacotherapy.

[3] Huber CG (2012). Brief Psychiatric Rating Scale—Excited Component (BPRS-EC) and neuropsychological dysfunction predict aggression, suicidality, and involuntary treatment in first-episode psychosis. Schizophrenia Research.

[4] Huber CG (2014). Evidence for an agitated–aggressive syndrome predating the onset of psychosis. Schizophrenia Research.

[5] Huber CG (2016). Evidence for an agitated-aggressive syndrome in early-onset psychosis correlated with antisocial personality disorder, forensic history, and substance use disorder. Schizophrenia Research.

[6] Fusar-Poli P (2012). Neuroanatomical maps of psychosis onset: voxel-wise meta-analysis of antipsychotic-naive VBM studies. Schizophrenia Bulletin.

[7] Naudts K, Hodgins S (2006). Neurobiological correlates of violent behavior among persons with schizophrenia. Schizophrenia Bulletin.

[8] Hoptman MJ, Antonius D (2011). Neuroimaging correlates of aggression in schizophrenia: an update. Curr. Opin. Psychiatry.

[9] Soyka M (2011). Neurobiology of aggression and violence in schizophrenia. Schizophrenia Bulletin.

[10] Barkataki I (2006). Volumetric structural brain abnormalities in men with schizophrenia or antisocial personality disorder. Behav. Brain Res..

[11] Kumari V (2013). Reduced thalamic volume in men with antisocial personality disorder or schizophrenia and a history of serious violence and childhood abuse. European Psychiatry.

[12] Yang Y (2010). Reduced hippocampal and parahippocampal volumes in murderers with schizophrenia. Psychiatry Research: Neuroimaging.

[13] Puri BK (2008). Regional grey matter volumetric changes in forensic schizophrenia patients: an MRI study comparing the brain structure of patients who have seriously and violently offended with that of patients who have not. BMC psychiatry.

[14] Kumari V (2009). Dysfunctional, but not functional, impulsivity is associated with a history of seriously violent behaviour and reduced orbitofrontal and hippocampal volumes in schizophrenia. Psychiatry Research: Neuroimaging.

[15] Narayan V (2007). Regional cortical thinning in subjects with violent antisocial personality disorder or schizophrenia. American Journal of Psychiatry.

[16] Kumari V (2014). Lower anterior cingulate volume in seriously violent men with antisocial personality disorder or schizophrenia and a history of childhood abuse. Aus N Z J Psychiatry.

[17] Widmayer, S. *et al* Structural Magnetic Resonance Imaging Correlates of Aggression in Psychosis: A Systematic Review and Effect Size Analysis. *Frontiers in Psychiatry* **9**, 10.3389/fpsyt.2018.00217 (2018). 10.3389/fpsyt.2018.00217 PMC6000417 29930519

[18] Hoptman MJ (2005). Quantitative MRI measures of orbitofrontal cortex in patients with chronic schizophrenia or schizoaffective disorder. Psychiatry Research: Neuroimaging.

[19] Hoptman M (2006). Aggression and quantitative MRI measures of caudate in patients with chronic schizophrenia or schizoaffective disorder. The Journal of Neuropsychiatry and Clinical Neurosciences.

[20] Fusar-Poli P (2013). Progressive brain changes in schizophrenia related to antipsychotic treatment? A meta-analysis of longitudinal MRI studies. Neuroscience & Biobehavioral Reviews.

[21] Radua J (2012). Multimodal meta-analysis of structural and functional brain changes in first episode psychosis and the effects of antipsychotic medication. Neuroscience & Biobehavioral Reviews.

[22] Smieskova R (2012). Different duration of at‐risk mental state associated with neurofunctional abnormalities. A multimodal imaging study. Human Brain Mapping.

[23] Smieskova R (2012). Insular volume abnormalities associated with different transition probabilities to psychosis. Psychological Medicine.

[24] Lambert M (2008). Treatment of severe agitation with olanzapine in 166 patients with schizophrenia, schizoaffective, or bipolar I disorder. Pharmacopsychiatry.

[25] Soloff P, White R, Diwadkar VA (2014). Impulsivity, aggression and brain structure in high and low lethality suicide attempters with borderline personality disorder. Psychiatry Research: Neuroimaging.

[26] Lee TMC, Chan SC, Raine A (2008). Strong limbic and weak frontal activation to aggressive stimuli in spouse abusers. Molecular Psychiatry.

[27] Kumari V (2009). Neural and behavioural responses to threat in men with a history of serious violence and schizophrenia or antisocial personality disorder. Schizophrenia Research.

[28] Tikàsz A (2016). Anterior cingulate hyperactivations during negative emotion processing among men with schizophrenia and a history of violent behavior. Neuropsychiatric Disease and Treatment.

[29] Sigmundsson T (2001). Structural abnormalities in frontal, temporal, and limbic regions and interconnecting white matter tracts in schizophrenic patients with prominent negative symptoms. American Journal of Psychiatry.

[30] Van OJ, Linscott RJ, Myin-Germeys I, Delespaul P, Krabbendam L (2009). A systematic review and meta-analysis of the psychosis continuum: evidence for a psychosis proneness–persistence–impairment model of psychotic disorder. Psychological medicine.

[31] Eklund A, Nichols TE, Knutsson H (2016). Cluster failure: why fMRI inferences for spatial extent have inflated false-positive rates. Proceedings of the National Academy of Sciences.

[32] Fusar‐Poli P (2014). Evidence of reporting biases in voxel‐based morphometry (VBM) studies of psychiatric and neurological disorders. Human Brain Mapping.

[33] Riecher‐Rössler A (2007). The Basel early‐detection‐of‐psychosis (FEPSY)‐study–design and preliminary results. Acta Psychiatrica Scandinavica.

[34] Riecher-Rössler A (2009). Efficacy of using cognitive status in predicting psychosis: a 7-year follow-up. Biological psychiatry.

[35] Yung, A. R. *et al*. Prediction of psychosis: a step towards indicated prevention of schizophrenia. *The British Journal of Psychiatry* (1998). 9764121

[36] Yung AR (2004). Risk factors for psychosis in an ultra high-risk group: psychopathology and clinical features. Schizophrenia Research.

[37] Riecher-Rössler A (2008). The Basel Screening Instrument for Psychosis (BSIP): development, structure, reliability and validity. Fortschritte der Neurologie-Psychiatrie.

[38] Lehrl S, Triebig G, Fischer B (1995). Multiple choice vocabulary test MWT as a valid and short test to estimate premorbid intelligence. Acta Neurologica Scandinavica.

[39] Lindenmayer JP (2004). An excitement subscale of the Positive and Negative Syndrome Scale. Schizophrenia research.

[40] Andreasen, N. C. Scale for the Assessment of Negative Symptoms (SANS). *The British Journal of Psychiatry* (1989). 2695141

[41] Spitzer, R. L. *et al*. Global assessment of functioning (GAF) scale. *Outcome assessment in clinical practice* 76–78 (1996).

[42] Borgwardt SJ (2007). Regional grey matter volume abnormalities in the at risk mental state. Biological Psychiatry.

[43] Ashburner J (2007). A fast diffeomorphic image registration algorithm. Neuroimage.

[44] Evans, J. D. *Straightforward Statistics for the Behavioral Sciences*. Brooks/Cole (1996).

